# Drug Discovery for Histone Deacetylase Inhibition: Past, Present and Future of Zinc-Binding Groups

**DOI:** 10.3390/ph18040577

**Published:** 2025-04-16

**Authors:** Gustavo Salgado Pires, Heber Victor Tolomeu, Daniel Alencar Rodrigues, Lídia Moreira Lima, Carlos Alberto Manssour Fraga, Pedro de Sena Murteira Pinheiro

**Affiliations:** 1 Laboratório de Avaliação e Síntese de Substâncias Bioativas (LASSBio), Instituto de Ciências Biomédicas, Universidade Federal do Rio de Janeiro, Cidade Universitária, Rio de Janeiro 21941-902, Brazil; gusalpi1@hotmail.com (G.S.P.); heber-victor@hotmail.com (H.V.T.); lmlima23@gmail.com (L.M.L.);; 2 Programa de Pós-Graduação em Farmacologia e Química Medicinal (PPGFQM), Instituto de Ciências Biomédicas, Universidade Federal do Rio de Janeiro, Cidade Universitária, Rio de Janeiro 21941-902, Brazil; 3 School of Pharmacy and Biomolecular Sciences (PBS), Royal College of Surgeons in Ireland, 1st Floor Ardilaun House Block B, 111 St Stephen’s Green, Dublin 2, Ireland; danielalencar@rcsi.com; 4 Instituto Nacional de Ciência e Tecnologia de Fármacos e Medicamentos (INCT-INOFAR), Universidade Federal do Rio de Janeiro, Cidade Universitária, Rio de Janeiro 21941-902, Brazil

**Keywords:** HDAC selectivity, metal chelation, epigenetics, drug design, structure–activity relationship, metalloenzyme

## Abstract

Histone deacetylases (HDACs) are key regulators of gene expression, influencing chromatin remodeling and playing a crucial role in various physiological and pathological processes. Aberrant HDAC activity has been linked to cancer, neurodegenerative disorders, and inflammatory diseases, making these enzymes attractive therapeutic targets. HDAC inhibitors (HDACis) have gained significant attention, particularly those containing zinc-binding groups (ZBGs), which interact directly with the catalytic zinc ion in the enzyme’s active site. The structural diversity of ZBGs profoundly impacts the potency, selectivity, and pharmacokinetics of HDACis. While hydroxamic acids remain the most widely used ZBGs, their limitations, such as metabolic instability and off-target effects, have driven the development of alternative scaffolds, including *ortho*-aminoanilides, mercaptoacetamides, alkylhydrazides, oxadiazoles, and more. This review explores the structural and mechanistic aspects of different ZBGs, their interactions with HDAC isoforms, and their influence on inhibitor selectivity. Advances in structure-based drug design have allowed the fine-tuning of HDACi pharmacophores, leading to more selective and efficacious compounds with improved drug-like properties. Understanding the nuances of ZBG interactions is essential for the rational design of next-generation HDACis, with potential applications in oncology, neuroprotection, and immunotherapy.

## 1. Introduction

Epigenetics is the area of biology that studies changes in phenotype related to factors extrinsic to the DNA sequence that are inherited during cell division. The term was originally described by biologist Conrad Waddington in his study of cell differentiation in embryonic development at the molecular level [1]. Two antagonistic concepts have been associated with this phenomenon: phenotypic plasticity and canalization, the latter of which was also coined by Waddington. Phenotypic plasticity refers to the ability of a gene to produce different phenotypes, while canalization refers to the inherent stability of some phenotypes in the face of environmental changes [2,3]. In this way, a complex of regulatory processes capable of altering gene expression was suggested, resulting in changes in phenotype, but without mechanistic descriptions [4]. The term was later used by David Nanney, who attributed the modulation of the expression of specific genes to epigenetic mechanisms, including the concept of perpetuation of gene expression states during the process of cell division, establishing the basic principles of epigenetics [2]. Despite this, it was only in 2009 that the new operational and unified definition of epigenetics was suggested [5].

As studies in the field have progressed, new techniques have been developed in order to describe mechanisms that explain the phenomena observed, complementing the understanding of epigenetics. The main epigenetic modifications observed are post-translational modifications in histones (acetylation/deacetylation, methylation, phosphorylation, etc.), DNA methylation, and modulation by microRNAs (miRNA) [4,6,7].

miRNAs are non-coding RNAs with 18–25 nucleotides in a single strand and are capable of modulating gene expression at the post-transcriptional level. They are initially transcribed in the form of a primary miRNA (pri-miRNA) by RNA-polymerase II, with a 5′ cap and a 3′ poly-A tail similar to that of a messenger RNA (mRNA), in addition to performing intramolecular pairing between their nitrogenous bases, assuming a pattern known as hairpin [8]. These pri-miRNAs are processed by the microprocessor complex containing the specific enzyme Drosha, giving rise to the miRNA precursor (pre-miRNA). Subsequently, the pre-miRNA is transported from the nucleus to the cytoplasm where it is once again processed, giving rise to the single-stranded miRNA. The activity of miRNA is related to complementary recognition of a mRNA by pairing nitrogenous bases, silencing it or directing it to degradation [9].

DNA methylation is one of the most characterized epigenetic mechanisms for a wide variety of organisms and involves the direct alteration of DNA through the introduction of a methyl group, without altering the sequence of the nitrogenous bases themselves [10]. To do this, a methyl group present in *S*-adenosylmethionine (SAM) is transferred mainly to position 5 of the pyrimidine ring of cytosines, and is catalyzed by the DNA methyl transferases (DNMT) family of enzymes [11]. In most cases, this methylation occurs on cytosines followed by guanines separated by phosphodiester bonds, called CpG sites. These sites are present throughout the genome, mostly methylated, with the exception of CpG islands, which are regions with a high density of CpG sites and are often present in promoter regions and show low methylation. These unmethylated regions are capable of recruiting transcription factors and inducing a less condensed chromatin state [12]. However, when methylated, they are associated with promoter silencing and decreased expression, both by preventing their binding to transcription factors and potentially recruiting proteins associated with repression [11].

**Figure 1:**
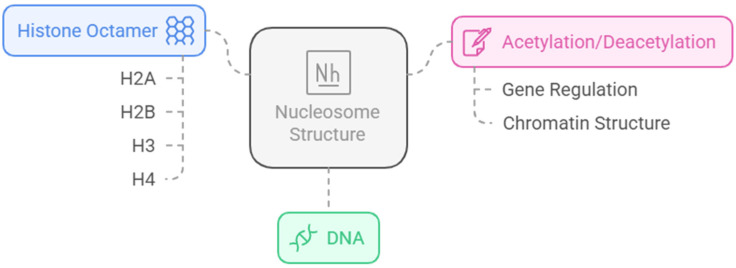
General composition and functions of a nucleosome, operating in gene regulation and chromatin structure [16]. Generated using Napkin AI tool, https://www.napkin.ai (accessed on 10 February 2025).

Finally, one of the most important post-translational modifications in histones is acetylation/deacetylation. However, in order to understand this type of modification, it is important to consider the general structure of the nucleosome (Figure 1). The nucleosome is a multiprotein complex present in all eukaryotic genomes and represents the fundamental unit of chromatin that repeats every 160–240 base pairs. It is made up of around 145–147 base pairs of DNA coiled by hydrogen bonds around histone cores, which are composed of two dimers of the histone proteins, H2A and H2B, and a tetramer of H3 and H4, making up an octameric structure [13,14,15].

**Figure 2:**
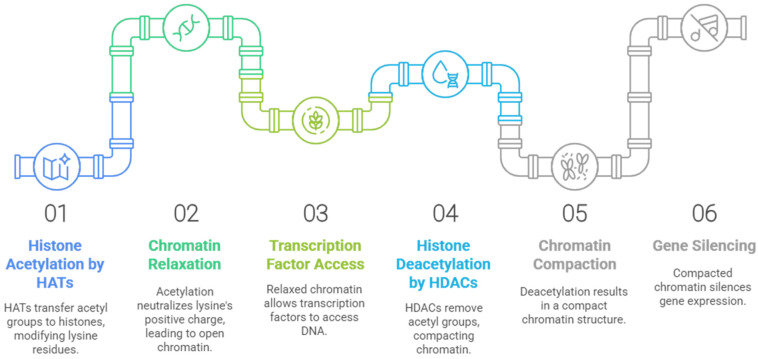
Dynamic process of epigenetic regulation, where epigenetic writers such as histone acetyltransferases (HATs) and histone methyltransferases (HMTs) mark amino acid residues, while epigenetic erasers such as histone deacetylases (HDACs) and lysine demethylases (KDMs) remove the markers, leading to activation or repression of gene transcription [21]. Generated using Napkin AI tool, https://www.napkin.ai (accessed on 10 February 2025).

In this context, the balance between acetylation and deacetylation of histones is carried out by two families of enzymes: histone acetyltransferases (HATs), which catalyze the transfer of an acetyl group from acetyl-CoA to lysine residues present in the *N*-terminal tails of histone proteins; and histone deacetylases (HDACs), which catalyze the reverse process (Figure 2). By altering the interactions of histones with DNA, the balance between histone acetylation and deacetylation can affect chromatin structure. The positive charge of the lysine residues in the ε-amino position is neutralized when acetylation occurs. This results in an open conformation of the chromatin, which allows access to specific transcription factors in addition to the transcription machinery. HDACs were initially associated with repressive activity, once the acetyl group removal by HDACs results in a compact chromatin that silences gene expression [17,18]. However, suppression of HDACs is also associated with the upregulation of some genes, especially by the acetylation of histones around their transcriptional start site. Thus, the role of HDACs in the balance of acetylation of histones results in both up- and downregulation, depending on the gene [19]. In addition, some HDACs have the ability to regulate non-histone proteins, such as cytoplasmic proteins and transcription factors [20].

Considering the interest in the discovery of new selective and efficacious HDAC-targeted compounds with improved drug-like properties, several previous reports address this issue [22,23,24]. However, due to limitations regarding the most common used pharmacophore, great effort has been made in the development of new drugs containing alternative scaffolds to circumvent this problem. In that sense, this review aims to address major limitations in the current literature, including the limited coverage of recently discussed Zinc-binding groups (ZBGs) and the insufficient discussion of DMPK properties, particularly in relation to these newer ZBGs.

## 2. Histone Deacetylases

There are eighteen HDACs identified in the human genome, divided into five classes:

- Class I: including HDACs 1, 2, 3 and 8;
- Class IIa: including HDACs 4, 5, 7 and 9;
- Class IIb: including HDACs 6 and 10;
- Class III: including sirtuins 1–7;
- Class IV: composed of HDAC11 as the only member [20,25].

These classes can be further divided into two large groups: the classic HDACs, which are those dependent on zinc ion (classes I, IIa, IIb, and IV) and the sirtuins, which are enzymes dependent on nicotinamide adenine dinucleotide (NAD^+^) (class III) [20,26,27].

Class I HDACs are HDACs 1, 2, 3, and 8, which are homologous proteins to the yeast Rpd3 protein and are expressed in various tissues and cells. Of these, the first three isoforms are nuclear, while HDAC8 is both nuclear and cytoplasmic [25,28].

HDACs 1, 2, and 3 make up multiprotein complexes that participate in epigenetic control by interacting with specific DNA sequences, leading to repression of the transcription of the corresponding genes. These complexes are formed by association with co-repressor proteins, such as switch intensive 3 (Sin3), nuclear receptor co-repressor (N-CoR), nucleosome remodeling deacetylase (NuRD), co-repressor of RE1 transcription (CoREST) or silencing mediator of retinoic acid and thyroid hormone receptors (SMRT) [20,25,28].

HDAC8, however, is considered an atypical member of the HDAC family due to its tissue-specific expression. Lacking a C-terminal region (responsible for recruiting multiprotein complexes and determining their localization) HDAC8 is not associated with any complex, and its activity has been attributed to its uncomplexed polypeptide form. Its activity can also be regulated through phosphorylation by protein kinase A (PKA) [20,25,28,29].

Class II HDACs are homologous to the yeast Hda1 protein, and can be further subdivided into classes IIa and IIb according to the presence or absence of a double deacetylase domain [25].

Class IIa, which includes HDACs 4, 5, 7, and 9, has only one deacetylase domain in the *C*-terminal region and an adapter domain in the *N*-terminal region. Isoenzymes of this class play an important role in blocking muscle differentiation, since they have a conserved binding site for myocyte enhancer factor 2 (MEF2) and, when bound, lead to inhibition of MEF2 [29]. In addition, their adapter domain has the function of regulating their access to the nucleus. This region contains serine residues which, when phosphorylated, allow them to associate with 14-3-3 proteins, leading to their retention in the cytoplasm and preventing their interaction with transcription factors [30]. The expression of isoenzymes of this class is tissue-specific, but variable for each one. All seem to be expressed in smooth muscle, but HDAC4 and 5 are also present in the brain and heart, with HDAC4 still participating in ossification; HDAC7 being found in endothelial cells, placenta, pancreas, and thymocytes; and HDAC9 being less present in the heart [20,31]. In addition, the deacetylase activity of HDAC9 is attributed to its association with HDAC3, which occurs through the co-recruitment of SMRT/NCoR co-repressors in the nucleus, not proving efficient in the absence of HDAC3 [28,32].

For class IIb HDACs, HDAC6 has a primarily cytoplasmic localization, with catalytic activity in non-histone proteins such as α-tubulins, tau, and heat shock protein 90 (HSP90) [28,30]. HDAC6 is directly related to cytoskeleton regulation, since it modulates microtubule acetylation levels. Its activity contributes to favoring cell mobility, since the hypoacetylated state of microtubules induces a more dynamic state, while acetylation makes them more stable [33]. In addition, the activity of this isoform in HSP90 is responsible for its regulation, where the deacetylation of this protein by HDAC6 allows the formation of the chaperone complex, favoring its activity [34]. HSP90 is essential for the formation of various client proteins in the appropriate conformation, and also acts to prevent the formation of aggregates of non-native proteins [35,36,37,38]. HDAC6 also has a zinc finger domain, which enables it to bind to ubiquitinated proteins, determining whether they will be degraded by the proteasome or whether they will accumulate in cells, forming aggresomes [39].

HDAC10, on the other hand, is related to HDAC6, but differs in the absence of the additional catalytic domain, but by associating with HDAC3, it is able to act on both nuclear and cytoplasmic substrates [25,40].

Class IV histone deacetylases have only one representative isoenzyme, HDAC11. It is mainly a nuclear isoform, but it is believed that it can act on non-histone proteins by associating with HDAC6. It is expressed particularly in the kidneys, brain, skeletal muscle and heart, and is structurally related to classes I and II [41]. Due to its more recent discovery, little is known about its physiological function or the therapeutic potential of its modulation. However, the isoform has been associated with immune regulation by blocking the expression of interleukin 10 (IL-10), and has also been attributed a fatty acid deacetylation activity. The role of its catalytic activity in these processes has yet to be elucidated [42,43].

Class III HDACs, also known as sirtuins, are homologous to the Sir2 protein of yeast, and are an atypical class that has not been considered as HDACs, since their deacetylase domain is only conserved within the seven members of the class (SIRT1-7), and they are not dependent on zinc, but on NAD^+^ [44]. Regarding their subcellular localization, it can be said that SIRT1, SIRT2, SIRT6, and SIRT7 are nuclear isoforms, with the SIRT1 and SIRT2 isoforms also being cytoplasmic, while SIRT3, SIRT4, and SIRT5 are mitochondrial isoforms [45]. They can also be divided into subclasses, with SIRT1, 2, and 3 being class I sirtuins, with deacetylase activity and SIRT4 class II, with the main activity of ADP-ribosyl transferase, as well as class III, composed only of SIRT5, showing primarily lysine demalonylase and desuccinylase activity, and class IV, made up of SIRT6 and 7, which show deacetylase activity in histones, as well as activities such as desuccinylase or in fatty acids [46,47,48,49]. Given the great differences in their activity and the structure of their deacetylase domain, only the classic HDACs will be discussed for selectivity evaluation.

### 2.1. HDACs as Therapeutic Targets

#### 2.1.1. HDACs in the Context of Cancer

HDACs are often correlated with the process of tumorigenesis, since they are related, in many cases, to the regulation of gene expression. Although somatic mutations are uncommon in these proteins, alterations in their expression or in the regulation of their genes can lead to a deregulation of their activity, favoring the development of tumors [50].

One of the possible reasons for this correlation is modifications in histone H4. An alteration present only in cancer cells is the loss of mono-acetylation at lysine 16 and trimethylation of lysine 20, which is associated with hypoacetylation resulting from exacerbated HDAC activity. Over-expression of HDAC can also be seen in various cancer cells, with this alteration having been recorded in stomach, colon, and breast cells [51].

HDACs can also be recruited by chimeric proteins formed by chromosomal translocations, affecting gene transcription. An example of this is the translocation between the promyelocytic leukemia protein (PML) and the retinoic acid receptor α (RARα), leading to the formation of PML-RARα. This chimeric protein is capable of recruiting multiprotein complexes in which HDAC is present due to its affinity for co-repressor proteins [51,52]. Under normal conditions, RARα is a nuclear receptor which, upon binding with retinoic acid, acts as a transcription repressor, regulating myeloid differentiation. The PML-RARα fusion protein, however, has the characteristic of losing sensitivity to retinoic acid by binding to complexes containing chromatin-modifying enzymes, such as HDAC [52,53]. Another example of this chimeric protein formation by chromosomal translocation is PLZF-RARα (fusion protein of the zinc finger of the promyelocytic leukemia protein with RARα). In both cases, there is a correlation with the development of promyelocytic leukemia [51,53].

Increased expression and activity of classical HDACs is a common alteration in various types of cancer, and most of them have been correlated with the progression of the disease, the type of cancer, and its prognosis according to the isoform in question [50]. However, among the isoenzymes, class I HDACs are the most commonly associated with cancer development, which is to be expected, since they have physiological functions related to survival, differentiation, cell cycle progression, and cell proliferation, as well as being nuclear localized [54]. Thus, epigenetic modulation by inhibiting HDACs results in the arrest of these processes, culminating in apoptosis, and is of interest for cancer treatment [21,51,55,56].

#### 2.1.2. HDACs in the Context of Neurodegenerative Diseases

Although it was initially aimed at treating cancer, HDAC inhibition was later shown to have potential in the treatment of neurodegenerative diseases such as Alzheimer’s disease (AD), Huntington’s disease (HD), Parkinson’s disease (PD), and multiple sclerosis [57,58,59,60,61].

HDAC inhibitors (HDACi) are an interesting alternative for the treatment of neurodegenerative diseases, as they have shown results in increased neuroplasticity, learning, memory, and neuroinflammatory effects. Even after neuronal loss, the increase in acetylation levels in histone H3 and H4 proteins provided by HDAC inhibition showed an increase in the number of synapses and recovery of learning and memory [62,63,64]. This effect was observed especially with HDAC2 inhibition and seems to be related to an increase in the density of dendritic spines, which are small protrusions present in the dendrite membrane of excitatory neurons, acting as receptive areas in the postsynaptic compartment of glutamatergic synapses. This increase in the density of dendritic spines associated with an increase in the levels of acetylation of histone H3 and H4 proteins seems to be associated with greater activation of the CREB-CBP [65,66,67]. In the main signal transduction pathway described, cyclic adenosine monophosphate (cAMP) induces the phosphorylation of the transcription factor CREB (pCREB), catalyzed by protein kinase A (PKA), thereby activating it. In turn, pCREB recruits CREB-binding protein (CBP), a transcriptional coactivator with HAT activity, and binds to cAMP response elements (CRE), modulating transcription [67,68]. HDAC6 is also a possible therapeutic target to be explored in the treatment of neurodegenerative diseases such as AD and other disorders related to the Tau protein [57,69]. This is due to the unique characteristics of HDAC6 in relation to other enzymes in its family, since it has a mainly cytoplasmic localization and two deacetylase domains. Among the non-histone proteins that are affected by HDAC6 is the microtubule-binding protein Tau, which apparently undergoes deacetylation of the Lys321 residue, favoring the phosphorylation of the Ser324 residue by kinases such as glycogen synthase kinase 3β (GSK3β), which contributes to the hyperphosphorylation of Tau. In this way, Tau is inactivated, losing its ability to bind to microtubules and stabilize them, consequently leading to cumulative aggregation and resulting in the formation of neurofibrillary tangles [70]. It was also observed that the selective inhibition of HDAC6 was able to both inhibit the action of β/γ-secretases, which are responsible for the formation of the β-amyloid peptide (Aβ) by cleaving the β-amyloid precursor protein (APP) and promote the autophagy of Aβ in vivo [71]. In addition, the relationship between HDAC6 and the regulation of HSP90 activity is also of interest in the context of neurodegenerative diseases. Despite the physiological importance of HSP90 in the correct folding of client proteins, which is favored by its deacetylated form, the hyperacetylation resulting from HDAC6 inhibition contributes to the reduction of Tau levels, since this form leads to the degradation of the client protein [34,69,72,73].

### 2.2. Structural Details of HDAC Isoforms

**Figure 3:**
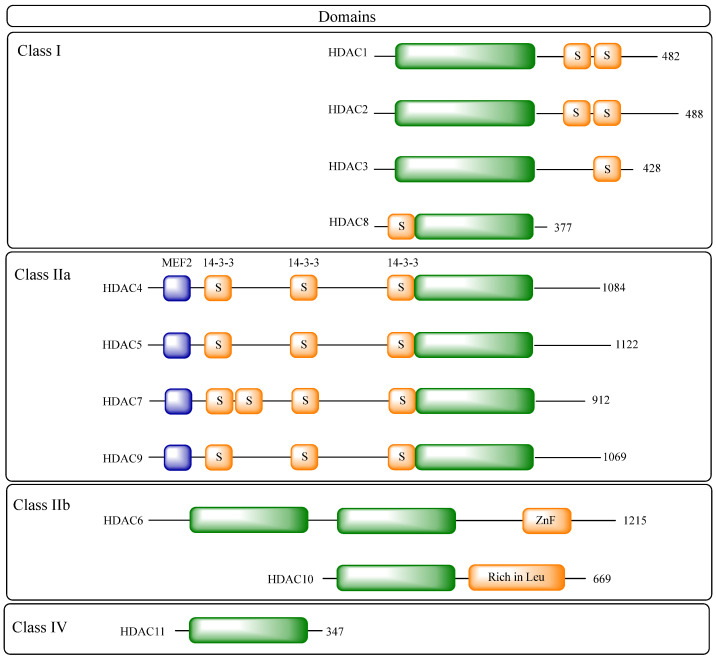
The histone deacetylase family (HDACs) and their main domains. Green rectangles represent the highly conserved deacetylase domain, blue rectangles represent myocyte enhancer factor 2 (MEF2) binding sites, yellow rectangles represent 14-3-3 protein binding sites, followed by the total amino acid count of the isoform. Adapted from [77].

The histone deacetylase family plays a fundamental role in epigenetic control and homeostasis, and is present in various organisms, from mammals to plants and prokaryotes. Among the zinc-dependent HDACs, the deacetylase domain is highly conserved (Figure 3). The structure of the domain consists of a central β-sheet surrounded by a set of α-helices and loops that make the connection, with many of these loops contributing to the formation of the catalytic site containing the catalytic zinc ion [74,75,76].

Despite this high conservation, the selective inhibition of HDAC isoforms is of great interest and, to this end, some differences in the catalytic site can be explored [77]. One example is the difference in the dimensions of the channels of class I and class II isoenzymes, with the channel of a class I HDAC being narrower and deeper than that of class IIb [78].

**Figure 4:**
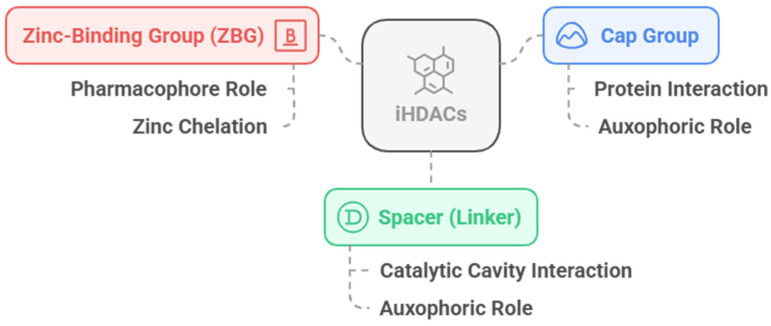
Representation of the classic pharmacophore model of HDACi, composed of the Cap group (blue), a linker (green) and the zinc-binding group (ZBG). Generated using Napkin AI tool, https://www.napkin.ai (accessed on 10 February 2025).

The HDACi have a well-characterized pharmacophoric features (Figure 4), with a zinc-binding group (ZBG) as the pharmacophoric group, a group for interaction with residues on the surface of the protein, the so called cap group, and a linker that connects these two subunits and interacts with the HDAC channel or gorge, with the latter two being responsible for auxophoric interactions [79].

**Figure 5:**
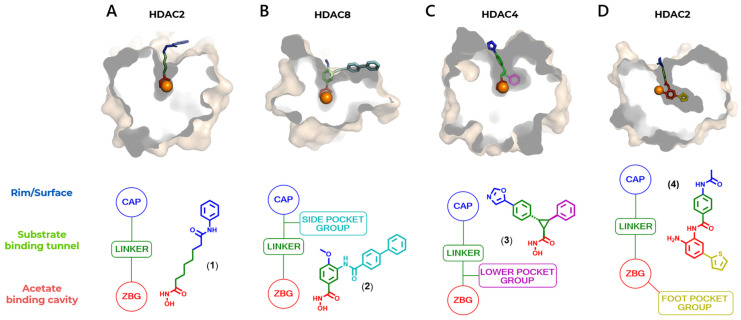
Cavities and subcavities of HDAC isoforms and their pharmacophore model. (**A**) Main cavity, composed of its surface (blue), substrate-binding channel (green) and acetate-binding cavity (red)—human HDAC2 cocrystallized with vorinostat (**1**); PDB: 4LXZ, with the pharmacophoric model, composed of the Cap group (blue), a linker (green), and the zinc chelating group (red). (**B**) Main cavity with side pocket (cyan)—HDAC8 from Schistosoma mansoni cocrystallized with TH65 (**2**); PDB: 6HTH. (**C**) Main cavity with lower pocket (magenta)—human HDAC4 cocrystallized with a cyclopropylhydroxamic acid derivative (**3**); PDB: 4CBY. (**D**) Main cavity with foot pocket (yellow)—human HDAC2 cocrystallized with a *p*-thienyl-anilinobenzamide derivative (**4**); PDB: 4LY1 [22].

In addition, HDAC isoforms differ in the presence or absence of certain cavities, as well as specific differences in non-conserved amino acid residues that allow specific interactions to be explored, and selectivity to be obtained between classes or isoforms of interest. The catalytic domain has a main cavity and sub-cavities (Figure 5 and Table 1) [80,81,82,83]. The main cavity is present in all HDAC isoforms and consists of the cavity surface, present in the outermost region of the site, the substrate-binding channel and the acetate-binding cavity, in the innermost region and close to the zinc ion. The sub-cavities, in turn, may or may not be present, being specific to each isoform (Table 1) and may be in the open or closed state depending on the interaction with the ligand and the isoform. The sub-cavities identified for HDACs consist of the side pocket and lower pocket, which is often also referred to as the selectivity cavity, and the foot pocket [84,85]. The three-dimensional structures of HDACs can be found solved experimentally in the Protein Data Bank (PDB), co-crystallized with the respective appropriate ligands in order to explore interactions with the specific sub-cavities, these being suberoylanilide hydroxamic acid SAHA or vorinostat (**1**), in the 4LXZ crystal, TH65 (**2**) in the 6HTH crystal, cyclopropylhydroxamic acid derivatives, such as **3** (PDB: 4CBY) and p-thienyl-anilinobenzamide derivatives, such as **4** (PDB: 4LY1) (Figure 5).

### 2.3. Initial Assessment of Selectivity

As mentioned above, the catalytic site is considerably conserved within classical HDACs. However, differences related mainly to residue exchange can be exploited, which limits access to sub-cavities, as well as differences in the dimensions of the channel [78,80,81,82,83].

#### 2.3.1. Selectivity for Class I

**Figure 6:**
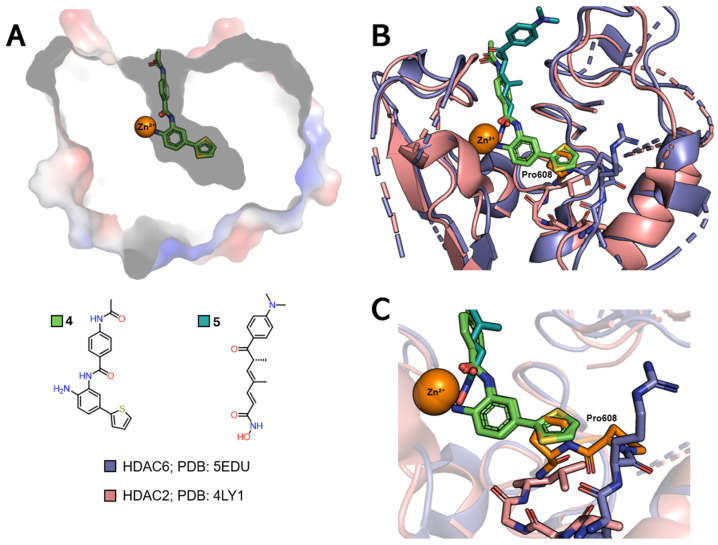
(**A**) Contour of the foot pocket subcavity of the PDB 4LY1 crystal cocrystallized with 4-acetamido-*N*-(2-amino-5-(thiophen-2-yl)phenyl)benzamide **4**; (**B**) superimposition of the PDB 5EDU (HDAC6) and 4LY1 (HDAC2) crystals, highlighting the Pro608 residue of HDAC6; (**C**) superimposition of the crystals in magnified view.

The selective inhibition of class I HDACs is of interest in the search for new treatments for diseases related to the regulation of gene transcription, such as cancer and viral diseases [23,54]. The HDAC channel in this class is narrower and deeper than those of class IIb HDACs [78]. In addition, inhibitors functionalized with the *ortho*-aminoanilide ZBG group can favor the selective inhibition of HDACs 1, 2, and 3 over other isoforms [78,79,86,87]. The insertion of a linking group into the foot pocket, usually characterized by aromatic rings such as 5-phenyl or 5-thienyl substituents, can lead to greater selectivity for isoforms 1 and 2, since it allows the foot pocket to be occupied and interactions to be explored in this region (Figure 6A) [22,23,81,88].

Comparing the PDB 4LY1 (HDAC2) structure, which features a foot pocket, and is complexed with the *p*-thienyl-anilinobenzamide derivative **4**, to the 5EDU (HDAC6) structure, co-crystallized with trichostatin A (**5**) and characterized by a straight channel with no subcavities (Figure 6B), reveals variations in the position of the loops near the region that grants access to the foot pocket. Additionally, the substitution of Gly143 in HDAC2 with a bulkier Pro608 residue in HDAC6 blocks access to this subcavity.

**Figure 7:**
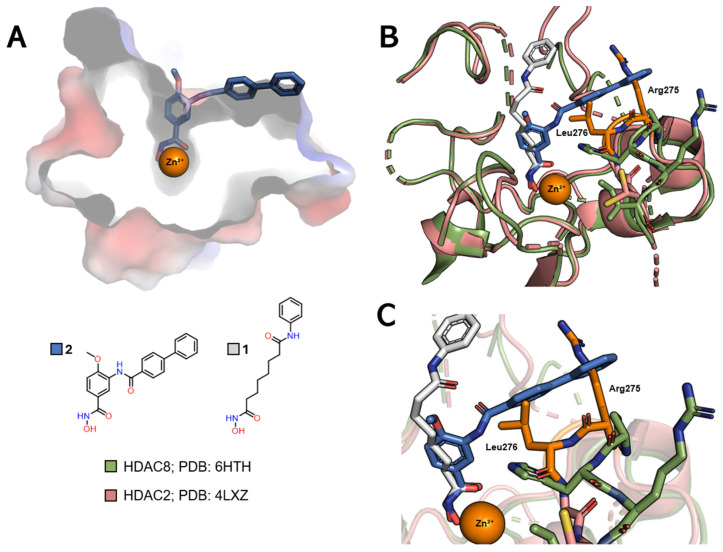
(**A**) Contour of the side pocket subcavity of the PDB 6HTH crystal co-crystallized with TH65 (**2**); (**B**) superimposition of the PDB 6HTH (HDAC8) and 4LXZ (HDAC2) crystals, highlighting the Arg275 and Leu276 residues of HDAC2; (**C**) superimposition of the crystals in magnified view.

HDAC8, on the other hand, differs from the other class I isoforms in that it has no foot pocket. This isoform does, however, have its own sub-cavity called the side pocket, close to the channel entrance, which can also be exploited for greater selectivity by adding a linker group to the side pocket, positioned on the linker (Figure 7A) [22,23]. In this case, the PDB 4LXZ (HDAC2) structure can be compared with the PDB 6HTH (HDAC8) structure, where it can be seen that HDAC8 co-crystallized with TH65 (**2**) also shows a variation in the position of the loops on the enzyme surface, which lead to the formation of the side pocket subcavity (Figure 7B). Of particular note is the alteration of a pair of residues, Arg275 and Leu276 in HDAC2, which is equivalent to Pro291 and His292 in HDAC8.

#### 2.3.2. Selectivity for Class IIa

**Figure 8:**
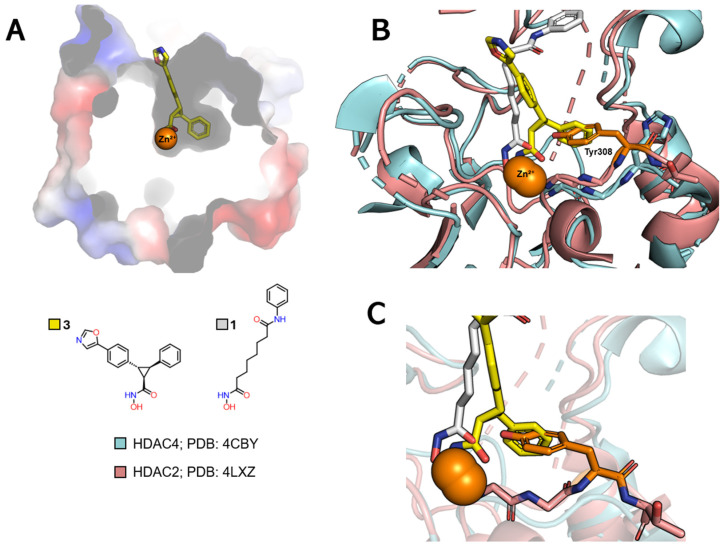
(**A**) Contour of the lower pocket of the PDB 4CBY crystal cocrystallized with (1R,2R,3R)-*N*-hydroxy-2-(4-(oxazol-5-yl)phenyl)-3-phenylcyclopropane-1-carboxamide **3**; (**B**) overlay of PDB crystals 4CBY (HDAC4) and 4LXZ (HDAC2), with emphasis on the Tyr308 residue of HDAC2; (**C**) overlay of crystals in magnified view.

Selective inhibition of class IIa HDACs, in turn, can be achieved by exploiting interactions in a third subcavity, called the lower pocket, but also referred to as the selectivity subcavity, which is adjacent to the main channel (Figure 8A) [84,89,90].

When comparing the PDB 4LXZ structure (HDAC2) with the PDB 4CBY structure (HDAC4), the latter being cocrystallized with the cyclopropylhydroxamic acid derivative **3**, one can observe the exchange of a Tyr308 in HDAC2 for a His976 in HDAC4 (Figure 8B). This can be considered in the design of selective ligands for HDACs of this class by adding a linking group that inserts into the lower pocket.

#### 2.3.3. Selectivity for Class IIb

The selective inhibition of class IIb HDACs is of interest since it has potential for application in the treatment of neurodegenerative diseases, especially regarding HDAC6. In part, this interest is due to the preferential cytoplasmic location of this isoform, which allows it to deacetylate non-histone targets, as well as giving it a low toxicity profile compared to class I HDACs, as they are preferentially located at the nuclear level [23,61].

When discussing the selectivity of HDAC6, it is important to consider the dimensions of the channel, which is wider and shorter than those of class I HDACs, with the exception of HDAC8. Thus, compounds with shorter and bulkier linkers, such as compounds with aromatic subunits in this region, tend to favor selectivity for isoform 6, as well as the option for bulkier and more extensive Cap groups [26,78]. Despite this, HDAC8 shows greater structural similarity in the main channel with HDAC6 compared to other class I isoforms, which makes selective inhibition of isoform 6 more difficult, especially considering that there is no subcavity identified near the HDAC6 channel [23].

**Figure 9:**
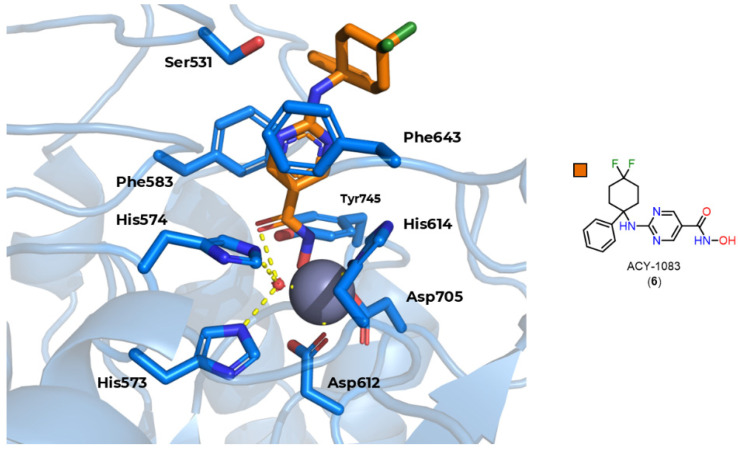
HDACi, ACY-1083 (**6**) (orange), cocrystallized with HDAC6 (blue). Coordination with the Zn^2+^ ion (gray) is represented by continuous lines, while hydrogen bonds are represented by dashed lines. View favoring interaction of the amino group of ACY-1083 (**6**) with the Ser531 residue [91].

However, there are still other characteristics that can be exploited to obtain selectivity for HDAC6, in particular the 531 serine residue present at the entrance to channel [91], which is capable of hydrogen bonding, and the use of aromatic rings in the linker, with the phenyl linker being the most described, allowing π–π type interactions with Phe583 and Phe643 present in the channel (Figure 9) [26,92,93].

## 3. Structure–Activity Relationship of HDAC Inhibitors

As already discussed, the classic pharmacophoric model of HDACi is characterized by the presence of a Cap group, a linker and a ZBG, and this structure is susceptible to the addition of other subunits for interaction with specific sub-cavities [22,79]. This topic will discuss the SAR related to each component of the pharmacophoric model.

### 3.1. Cap Group

The Cap group recognizes the protein surface through interactions with surface residues, primarily involving the L1 and L2 loops, with L1 traditionally responsible for most of the interactions with the Cap groups of HDACi.

Modifying the Cap group can be advantageous, as it may enhance affinity for different HDAC isoforms by optimizing surface interactions at the entrance of the main channel. Additionally, these modifications can promote selectivity for specific isoforms based on the relationship between the Cap’s volume and the dimensions of the target channel [79,94]. The greatest potential for modifications to this subunit lies in the structural diversity of the surface surrounding the HDAC channel, which is less conserved between isoforms. However, the high flexibility and large contact surface of this region promote favorable interactions between the Cap group and various HDACs, limiting selectivity—particularly when the Cap groups themselves are also flexible [22].

**Figure 10:**
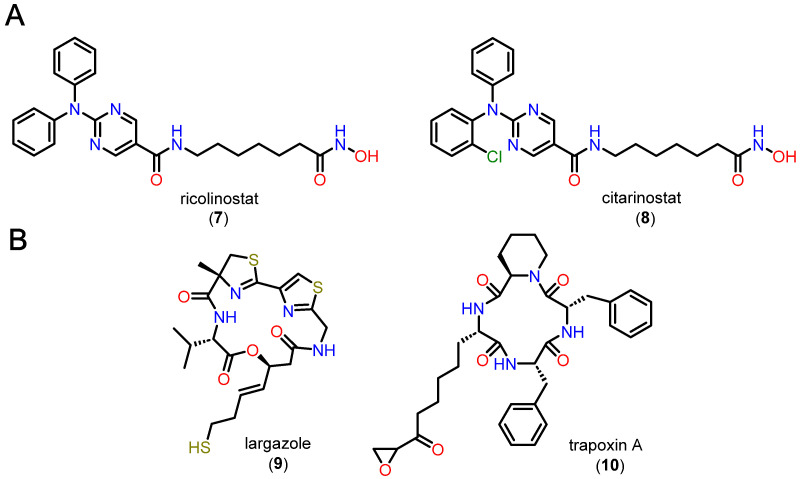
(**A**) Chemical structure of ricolinostate (**7**) and cytarinostate (**8**), drugs with bifurcated Cap groups. (**B**) Chemical structure of largazole (**9**) and trapoxin A (**10**), compounds with macrocycles as Cap groups.

Cap groups can be bifurcated, i.e., with two substituents, one binding to the L1 loop and the other concomitantly allowing interactions with L2. Another aspect related to bifurcated Cap groups is that there is an increase in volume compared to non-bifurcated groups. Bifurcated Cap groups that are bulky and sterically complementary to the surface of the channel entrance are possible alternatives for obtaining affinity and selectivity in HDAC6, whether or not they exploit hydrogen bonds accessible in this region [95]. Two examples of bifurcated Cap groups are ricolinostate (**7**) and cytarinostate (**8**), which are HDACi showing greater selectivity for isoform 6 (Figure 10A). Alternatively, compounds with Cap groups composed of macrocycles with selectivity for class I HDACs, especially HDAC1, have also been described, as in the case of largazole (**9**) and trapoxin A (**10**) (Figure 10B) [22,23].

It is also worth noting that the Cap group functions as an auxophoric group—meaning it does not participate in essential interactions for HDAC biological activity. This opens the possibility of incorporating pharmacophoric subunits targeting other proteins, paving the way for the design of novel multitarget ligands by molecular hybridization [23,96].

### 3.2. Linker

The linker is a subunit present in HDACi that connects the Cap group and the ZBG, as well as interacting with the cavity channel. Because of the differences in volume and interactions that can be exploited in different classes and isoforms of HDACs, alterations to this subunit are widely used in the structural design of selective inhibitors [26,78,79].

In this context, alkyl and vinyl linkers are well accepted by all HDAC isoforms, but with low isoform selectivity. It is important to note that compounds with this type of linker can still achieve selectivity depending on the Cap and ZBG group. Considering HDAC6, selective inhibition can still be achieved with a bulky Cap, although it is a less selective inhibition than with an appropriate linker [22,26]. In contrast, bulkier linkers, especially aromatic ones, show a selectivity profile for isoforms 6 and 8. The main example is the phenyl linker, which has a larger volume than those previously mentioned, as well as the possibility of π–π interactions [22,26,91]. However, aromatic heterocycles have been cited as an alternative for inhibiting HDAC6 with a better selectivity profile compared to HDAC8, such as the pyrimidine linker or five-membered cycles such as thiazole, oxazole, and oxadiazole [97].

### 3.3. Zinc-Binding Group (ZBG)

ZBG, the pharmacophoric group of HDACi, related to binding to the Zn^2+^ ion present in the catalytic site of classical HDACs and playing a fundamental role in the deacetylase mechanism of these enzymes [22,23,42,79,98]. It is present in the vast majority of inhibitors and contributes to their affinity for the target and, in some cases, can confer selectivity for specific isoforms [22,23].

**Figure 11:**
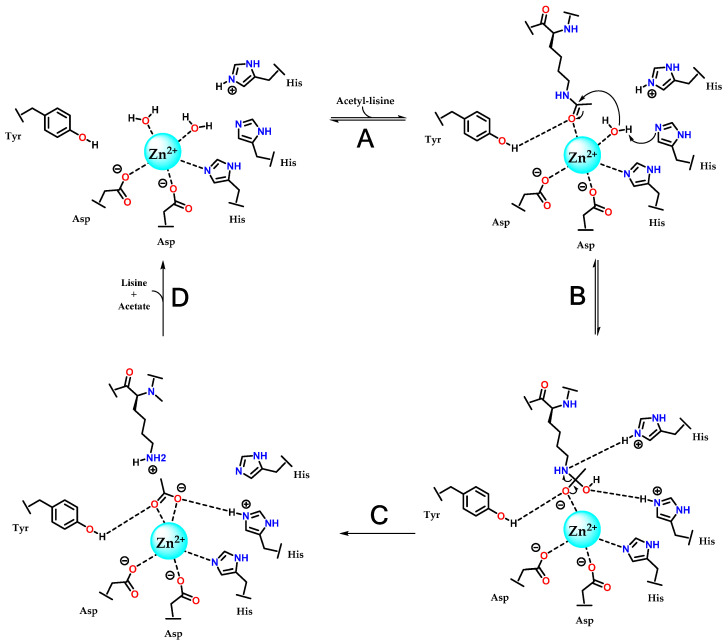
Proposed mechanism for deacetylation by classical HDAC isoforms. (**A**) Recognition of the acetylated lysine at the active site displaces a water molecule. (**B**) Formation a tetrahedral intermediate. (**C**) Completion of the acetylated lysine hydrolysis into lysine and acetate. (**D**) Return to its initial. Adapted from [75].

The catalytic mechanism of deacetylation by HDACs was first proposed with the determination of the crystal of a homolog in *Aquifex aeolicus*, a hyperthermophilic bacterium, which were called HDLP (histone deacetylase-like protein) and cocrystallized with vorinostat (**1**) and TSA (**5**). This model indicated the coordination of Zn^2+^ with two aspartate residues and one histidine residue, as well as the participation of two more histidines and one tyrosine [23,99]. This mechanism was later refined based on the structure of HDAC8, and is now considered to be the same for the other isoforms (Figure 11) [75]. The proposed mechanism is hydrolysis, in which zinc is initially coordinated with a histidine and two aspartates, in addition to two water molecules present in the catalytic site. The presence of the acetylated lysine at the active site displaces a water molecule, allowing the acetylated residue to coordinate with Zn^2+^ through the carbonyl group of the acetyl moiety. This also induces a conformational change in a tyrosine residue, which now acts as a donor in a hydrogen bond interaction with the same carbonyl (Figure 11A). A histidine residue abstracts a hydrogen from a water molecule coordinated to Zn^2+^, enabling it to perform a nucleophilic attack on the carbonyl, shifting the π electrons to the carbonyl oxygen and forming a tetrahedral intermediate (Figure 11B). The amino group of lysine, by abstracting the hydrogen from the protonated histidine residue, becomes a good leaving group, completing the hydrolysis into lysine and acetate (Figure 11C). Once lysine and acetate leave the active site, the enzyme returns to its initial state and can accept new substrates, completing its catalytic cycle (Figure 11D).

**Figure 12:**
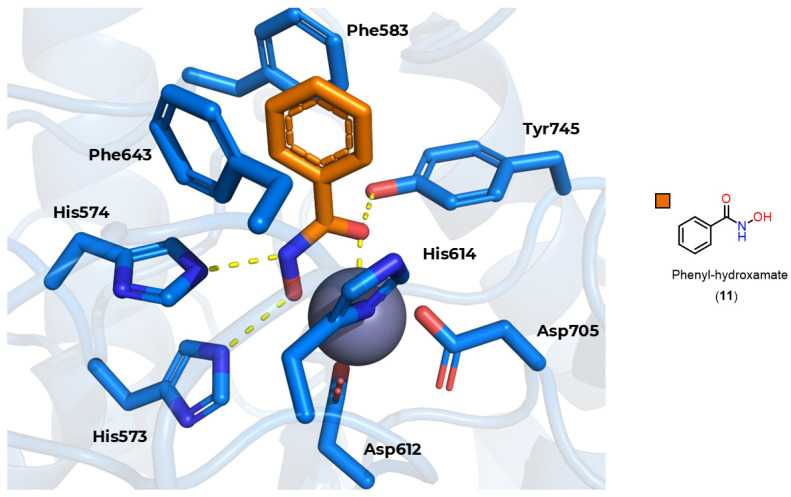
Phenylhydroxamate (**11**) without Cap group cocrystallized with HDAC6, allowing observation of the bidentate bonding mode mimicking the tetrahedral intermediate described in the HDAC deacetylation mechanism. Adapted from [101].

**Figure 13:**
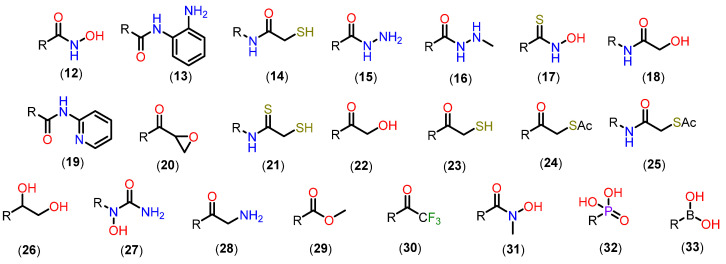
Chemical structure of functional groups that can function as ZBG [102,103].

Based on this mechanism, the first HDAC inhibitors exploit interactions in the active site by mimicking the tetrahedral intermediate, with hydroxamic acid emerging as a privileged scaffold (Figure 12). Classically, it is considered that the interaction with the Zn^2+^ ion takes place in a bidentate manner, which is indeed true in most cases. However, examples of monodentate interaction have also been reported. TSA (**5**) is able to coordinate with the Zn^2+^ ion in both a bidentate and monodentate way, in a ratio of 70:30, and with a free energy difference of 0.5 kcal/mol. This indicates that, although more unusual, there is no significant impairment of affinity with the enzyme [91,100]. This bond, however, is considered atypical, and could be explored especially in the case of HDAC6 with inhibitors with larger linkers, as in the case of phenylhydroxamate (**11**) and its derivatives [22,23,27]. Figure 13 shows functional groups that can function as ZBG, of which the first three stand out: hydroxamic acid (**12**), *ortho*-aminoanilide (**13**), and mercaptoacetamide (**14**).

#### 3.3.1. Hydroxamates

Hydroxamic acid (or hydroxamate) is the most classic and common ZBG due to its high affinity and ability to chelate the zinc ion, as well as being the best-studied functional group in this context and easy to obtain in synthesis [100,104,105]. Hydroxamates do not show selectivity for specific isoforms on their own, and are used in pan-inhibitors, although changes to the Cap group and linker can be strategies to confer selectivity [22,23].

**Figure 14:**
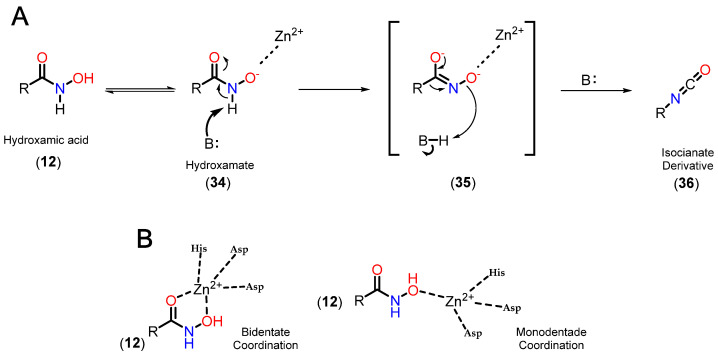
(**A**) Formation of isocyanate derivatives by Lossen rearrangement. (**B**) Form of monodentate and bidentate complexation of hydroxamates with Zn^2+^.

On the other hand, although few other ZBGs have comparable potency, it should be considered that hydroxamic acid, due to its great ability to coordinate with metals, is still capable of chelating the Zn^2+^ ion present in other zinc-dependent metalloenzymes, such as aminopeptidases, matrix metalloproteinases, and carbonic anhydrases [98]. Thus, another limitation is in terms of pharmacokinetic parameters, with high clearance and a short half-life, which can be explained by the possibility of hydrolysis, forming the respective carboxylic acids [106] which, in turn, can be targets for phase II metabolism. In addition, the effect of hydroxamate-induced genotoxicity and mutagenicity stands out, which is justified by the Lossen rearrangement (Figure 14) [24,107]. In this process, hydroxamic acid (**12**) is in equilibrium with its deprotonated form, hydroxamate (**34**), which can coordinate with Zn^2+^. This intermediate can then have its hydrogen captured by a base, displacing the π electrons from the carbonyl to the oxygen **35**. Subsequently, a π *C*=*O* bond is formed again, with the rearrangement of the R substituent, forming a new *C*-*N* bond and the elimination of the oxygen characteristic of hydroxamic acid, forming the respective isocyanate derivative (**36**), which are electrophilic and capable of reacting with DNA, causing toxicity [24,98,100,104,108].

#### 3.3.2. *Ortho*-aminoanilides

**Figure 15:**
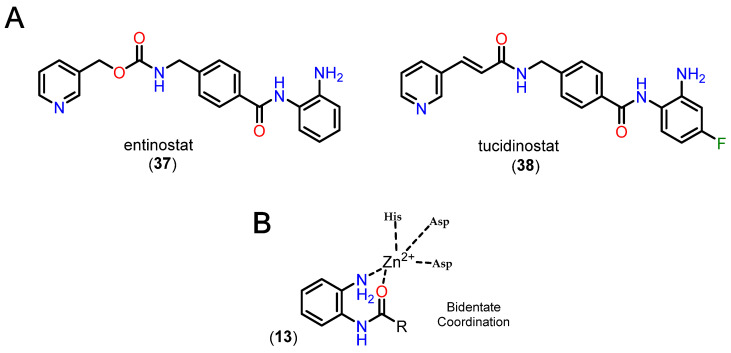
(**A**) Chemical structure of entinostat (**37**) and tucidinostat (**38**). (**B**) Form of bidentate complexation of *ortho*-aminoanilides with Zn^2+^.

Benzamides, especially *ortho*-aminoanilides, are one of the most prevalent and studied ZBGs in the context of HDACi. Compared to hydroxamic acid, *ortho*-aminoanilides show greater selectivity for class I HDACs, especially isoforms 1, 2 and 3 [105]. In addition, the group allows for even more substitutions on the aromatic ring, making it possible to add a linking group to the footpocket, especially with bulky groups in the para position, which confers greater selectivity for HDAC1 and 2 [22,86]. Two representatives of these inhibitors are entinostat (**37**), also known as MS-27-275, and tucidinostat (**38**) (Figure 15).

It is also reported that the *ortho* position of the amino group is fundamental for activity in HDACs, since the substituted entinostat analog did not show activity in HDACs or hyperacetylation in histones [109,110]. In addition, it is worth noting the longer residence time in the active site compared to hydroxamic derivatives [81]. Generally, *ortho*-aminoanilides chelate the Zn^2+^ ion in a bidentate manner through the nitrogen of the aniline and the oxygen of the carbonyl, while the aromatic ring protrudes towards the foot pocket, which justifies their selectivity profile [22,81,88,110].

#### 3.3.3. Mercaptoacetamides

Mercaptoacetamide is another ZBG used in HDACi, in this case because of its selectivity for HDAC6, resulting in HDACi with neuroprotective activity [24,111,112]. The main ZBG used for selective HDAC6 inhibition remains hydroxamic acid, despite its limitations in terms of pharmacokinetics and the risk of mutagenicity associated with the Lossen rearrangement. In this context, mercaptoacetamides are growing as a potential alternative for the selective inhibition of HDAC6, since they do not present the same risk of mutagenicity, which is of particular interest in the case of long-term treatments, such as in neurodegenerative diseases, as well as having suitable physicochemical properties with regard to the permeability parameter of the blood–brain barrier [24,110].

The first examples of the incorporation of mercaptoacetamides as ZBG in HDACi were reported in 2005, with the inhibition of total HDACs being observed, without distinguishing between isoforms [113,114]. Initially, molecular docking studies were carried out in order to define the binding modes of mercaptoacetamides with HDACs based on the PDB crystals of HDAC8 (PDB code 1T64, 1T67, 1T69 and 1VKG) [114], and also with a 3D model of HDAC1 obtained by homology based on the HDLP crystal (PDB code 1C3S) [113]. A bidentate mode of coordination with the Zn^2+^ ion was thus suggested via the deprotonated sulfur of the thiolate and the oxygen of the carbonyl.

**Figure 16:**
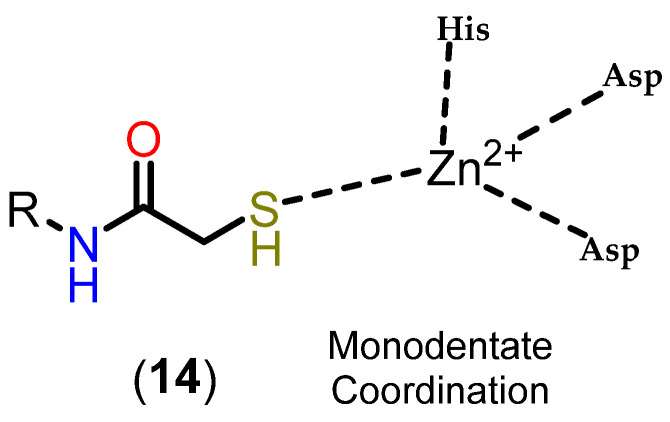
Form of monodentate complexation of mercaptoacetamides with Zn^2+^.

However, the compounds were subsequently tested again for inhibition of class I and IIb HDACs, with selectivity being observed for HDAC6 [24]. In addition, crystals of HDAC6 from Danio rerio (PDB code: 6MR5) and HDAC8 from Schistosoma mansoni (PDB code: 4CQF) cocrystallized with selective inhibitors for the respective isoforms featuring mercaptoacetamide as ZBG [112,115] are described. The crystals show that, contrary to what was previously described, coordination occurred in a monodentate manner in both cases (Figure 16). The tyrosine residue present in the catalytic site (Tyr745 in HDAC6 and Tyr341 in HDAC8) donates a hydrogen bond to the mercaptoacetamide carbonyl, while the deprotonated sulfur of the thiolate coordinates with the Zn^2+^ ion, as well as accepting a hydrogen bond from histidine residues in the active site (His573 in HDAC6 and His141 in HDAC8). It can also be seen that in the case of the HDAC6 crystal, unlike HDAC8, the mercaptoacetamide amide is able to donate a hydrogen bond to the histidine residue H574, which may contribute to the selectivity of this group for HDAC6 [112,115].

#### 3.3.4. Alkylhydrazides

**Figure 17:**
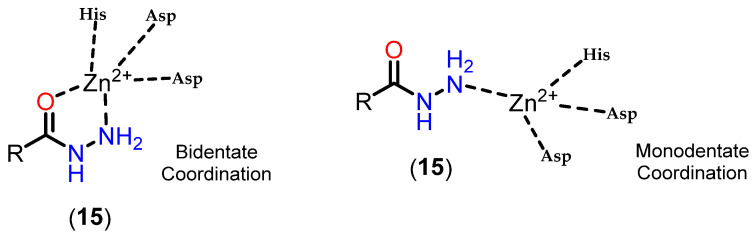
Form of monodentate complexation of hydrazides with Zn^2+^.

Another zinc-binding group that has emerged in several papers in the literature in the context of HDAC inhibition are alkylated hydrazides. The importance of hydrazide moieties in exhibiting potential HDAC inhibitory activity has been highlighted in numerous studies [116,117,118,119,120,121,122,123,124]. The hydrazide group, particularly at the terminal position, may act as an effective ZBG. Additionally, in the cap and linker regions, the hydrazide moiety can serve as a critical functional group. The interaction of alkylated hydrazides is analogous to that of hydroxamates when binding to Zn^2+^, occurring either in a bidentate or monodentate mode (Figure 17).

**Figure 18:**
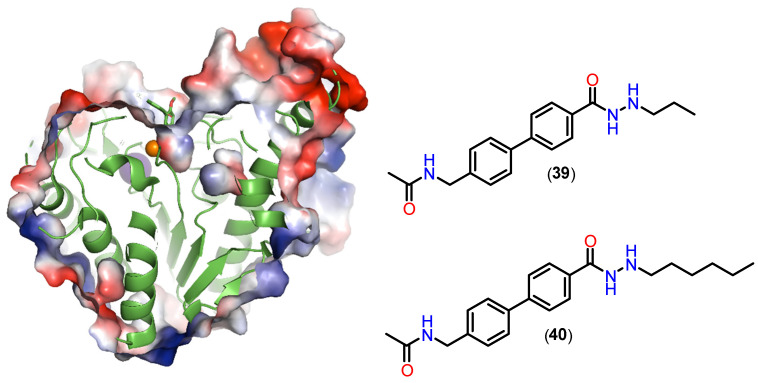
PDB:6HTH crystal cocrystallized with TH65 **2**, highlighting the isolated cavity near the active site channel. *n*-propyl (**39**) and *n*-hexyl (**40**) hydrazides from the work by Sun et al. [126].

Hydrazides with *n*-propyl or *n*-butyl side chains have been identified as novel HDAC3 inhibitors, demonstrating efficacy against acute myeloid leukemia both in vitro and in vivo [120,121,122,124,125]. Some of these compounds also exhibited favorable pharmacokinetic profiles. The hydrazide-based inhibitors are more bioavailable and stable compared to their hydroxamic acid-based counterparts, highlighting their potential for therapeutic application [125]. Alkylated hydrazides are versatile in terms of their selectivity for the different HDAC isoforms, and it is possible to target the inhibition based on the alkyl groups inserted. In the work by Sun and colleagues [126], it was observed that alkylated *n*-propyl hydrazides (**39**) showed selectivity for HDAC3, accommodating the propyl group in the foot pocket of this isoform. On the other hand, the *n*-hexyl derivative (**40**) surprisingly showed selectivity for HDAC8, exploiting a foot pocket that is not naturally present in HDAC8, but which can be accessed for this derivative. By analyzing the image (Figure 18), it is possible to see a probable foot pocket that is inaccessible to the cocrystallized inhibitor (**39**) (PDB: 6HTH), but which, in the case of the n-hexyl derivative (**40**), shows a communication between the channel and the isolated cavity.

#### 3.3.5. 5-(Difluoromethyl)-1,3,4-oxadiazole (DFMO)

**Figure 19:**
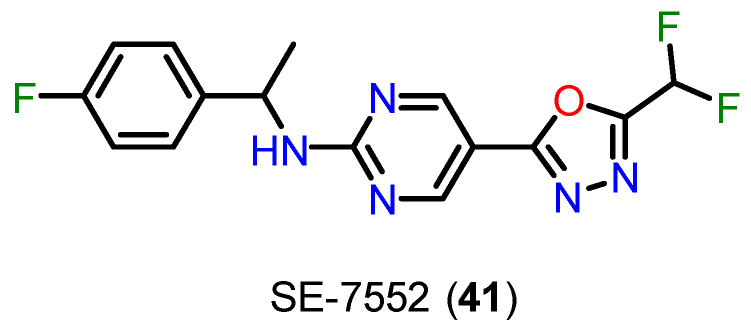
Chemical structure of SE-7552 (**41**).

Kim and coworkers have discovered the difluoromethyl-1,3,4-oxadiazole (DFMO) group as one of the most promising selective binding motifs for HDAC6 [127]. In their investigation, DFMO demonstrated strong selectivity over HDAC1 and excellent HDAC6 inhibition at low nanomolar doses [127]. Although frequently mentioned in patents [127,128,129,130,131,132], research articles comparatively underrepresent this ZBG. However, the DFMO derivative SE-7552 (**41**) (Figure 19) made its first appearance in scientific literature in 2022 when it was employed as a selective HDAC6 inhibitor to treat obesity-related leptin resistance [133]. The DFMO motif was successfully added to proteolysis-targeting chimeras (PROTACs) in 2022 in order to specifically degrade HDAC6 [134]. Despite these developments, it was still unknown how precisely DFMOs inhibit or degrade HDAC6. It is worth mentioning that the 5-(trifluoromethyl)-1,2,4-oxadiazole (TFMO) group has a bioisosteric relationship with DFMO [135,136].

It was discovered by Cragin and coworkers that an acyl hydrazide produced by an enzyme-catalyzed ring-opening reaction in a DFMO derivative co-crystallized in an extended arrangement within the HDAC6 active site [137]. Ptacek and colleagues [138] conducted a comparative evaluation of a hydroxamate-based HDAC6 inhibitor and its corresponding DFMO analog. Cell-based and biochemical tests clearly showed the DFMO ZBG’s excellent potency and selectivity [138]. Similarly, a recent patent revealed the great selectivity of DFMO-based HDAC6 inhibitors in comparison to a comparable hydroxamic acid-based HDAC6i [139].

Cellupica and colleagues [140] reported the structure of the HDAC6 complex with a hydrazide inhibitor formed through the double hydrolysis of a related oxadiazole inhibitor. The authors hypothesized that the substantial HDAC6 suppression shown might not be entirely due to the crystalline hydrazide. Rather, they suggested that there is a high-affinity intermediate that creates a tight, long-lasting enzyme-inhibitor combination. This intermediate might be a protonated acyl hydrazide or a closed hydrated intermediate, both of which are proposed as possible active species. Nevertheless, it was unable to definitively determine the precise nature of the active species [140].

**Figure 20:**
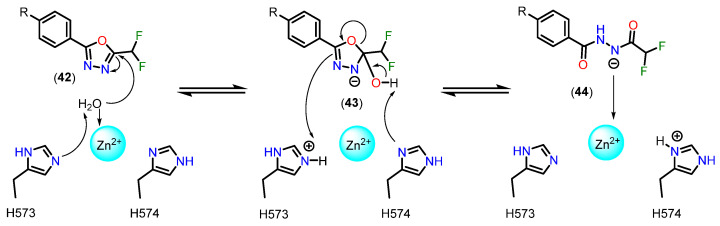
Proposed reaction mechanism of Zn^2+^ catalyzed ring opening reaction of DFMO derivative [141].

Cragin and coworkers [137] showed that DFMOs can inhibit HDAC6 via a two-step slow-binding process and function as selective, mechanism-based (Figure 20), and largely irreversible inhibitors. According to these results, the active species is a deprotonated difluoroacetyl hydrazide, which is produced when zinc-bound water attacks the sp^2^ carbon nearest the difluoromethyl part of the DFMO group. This is followed by oxadiazole ring-opening [141].

**Figure 21:**
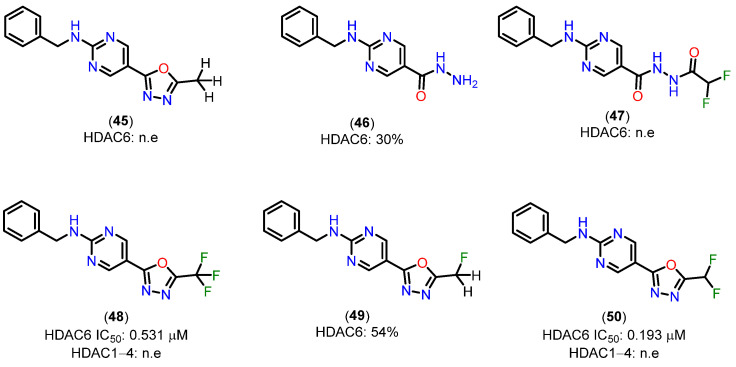
Structures of the methyl-1,3,4-oxadiazole **45**, hydrazide **46**, acylhydrazide **47**, trifluoromethyl-1,3,4-oxadiazole **48**, monofluoromethyl-1,3,4-oxadiazole **49** analogs and oxadiazole **50**. Inhibitory activities of prepared compounds against HDAC1–4 and HDAC6; IC_50_ [μM] or percent inhibition at 10 μM; n.e.: no effect = <15% inhibition at 10 μM [141].

To support the proposed mechanism above, the experimental results indicate that the C=N bond in the oxadiazole of compounds **45** and **49**, containing methyl and monofluoromethyl groups, is insufficiently activated for nucleophilic attack, preventing the formation of a zinc-bound nitrenium ion comparable to the deprotonated compound **47** [141]. The low inhibitory potency of compound **47** suggests that there is a higher energetic barrier to deprotonation and the subsequent formation of a zinc-bound nitrenium ion, as opposed to the hydrolysis of oxadiazole **50**, which would directly produce the zinc-bound nitrenium ion. The nitrenium forms a strong charge-charge interaction with the zinc ion, while the amino group in compound **46**, after deprotonation, would form a weaker charge–dipole interaction. This may explain the weaker inhibitory potency observed for compound **46** [141]. Structures of compounds **45**–**50** are shown in Figure 21.

## 4. Pharmacokinetic Profile

Drug pharmacokinetics and metabolism (DMPK) is a relevant area of pharmaceutical science. The approach to absorption, distribution, metabolism, and excretion (ADME) and pharmacokinetics (PK) research during drug discovery and evolution has changed in recent years, moving away from a predominantly descriptive approach towards a more quantitative and mechanistic understanding of the pathway of drug candidates in biological systems [142,143]. In the last decade, significant progress has been made not only in identifying the physicochemical characteristics of drugs that affect their ADME, target organ exposure and toxicity, but also in identifying design principles that can minimize potential drug–drug interaction (DDI) effects and reduce friction [142,143]. Table 2 presents an overview of the pharmacokinetic profile of main ZBGs further discussed in this section.

### 4.1. Hydroxamates Pharmacokinetic Profile

**Figure 22:**
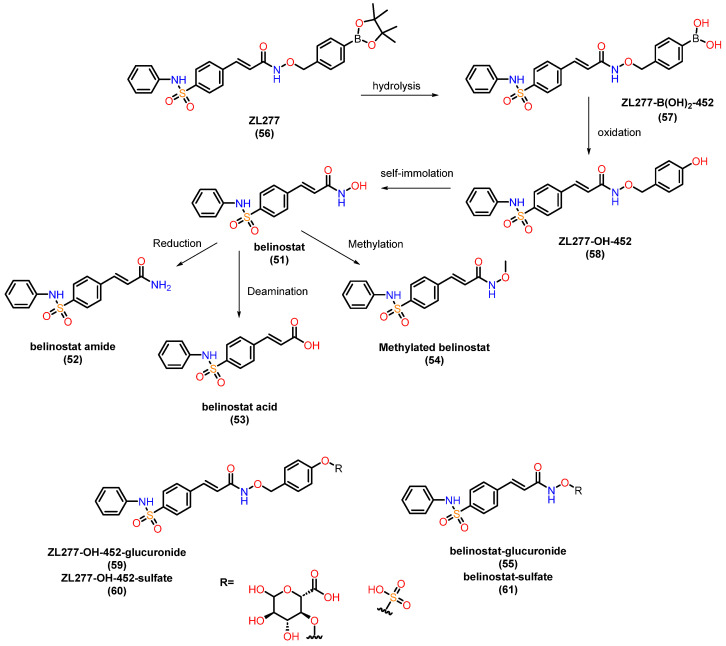
The metabolic pathways of ZL277 (**56**) and belinostat (**51**).

Belinostat (**51**) is the first of four HDAC inhibitors approved by the FDA to treat relapsed or resistant peripheral T-cell lymphoma [144]. The study of the pharmacokinetics (PK) and metabolism of belinostat (**51**) has been extensive [145,146,147,148,149,150]. Belinostat (**51**) undergoes accelerated glucuronidation, catalyzed by UGT1A1, -1A3, -1A8, -2B4, and -2B7 [145,146,150]. Glucuronidation is the main metabolic process of belinostat, mainly mediated by UGT1A1, and the predominant site of belinostat glucuronidation was found at the hydroxyl position. Other minor metabolites include belinostat amide (**52**), belinostat acid (**53**), belinostat methyl (**54**), and belinostat glucoside (**55**). These belinostat metabolites are inactive or very little active in clonogenic experiments. These observations contribute to understanding the low bioavailability and limited therapeutic efficacy of belinostat in animals. Zhang and colleagues [151] studied the in vitro metabolic profile (Figure 22) of ZL277 (**56**), a prodrug of belinostat, highlighting key metabolites: ZL277-B(OH)_2_-452 (**57**), the main oxidative metabolite ZL277-OH-424 (**58**), the active drug belinostat (**51**), as well as belinostat amide (**52**), belinostat acid (**53**), and methylated belinostat (**54**) in liver S9 fractions. Both ZL277-OH-424 (**58**) and belinostat (**51**) underwent glucuronidation in liver microsomes, while only ZL277-OH-424 (**58**), and not belinostat (**51**), underwent some degree of sulfation in rat liver cytosols. These metabolites were assessed in plasma and in an in vivo breast tumor model, as well as in urine and feces from mice treated with ZL277 (**56**). In the pharmacokinetic study of ZL277 (**56**), the active drug belinostat (**51**) showed a half-life (t_1/2_) of 10.7 h, an area under the curve (AUC) of 1506.9 ng/mL·h, and a maximum plasma concentration (C_max_) of 172 ng/mL, reached 3 h after a single dose of 10 mg/kg. The hydrolysis product of the prodrug, ZL277-B(OH)2-452 (**57**), showed an AUC of 8306 ng/mL·h and a C_max_ of 931 ng/mL, also reached 3 h after administration. The pharmacokinetics of ZL277 (**56**) demonstrated significantly higher bioavailability compared to belinostat (**51**).

### 4.2. Non-Hydroxamates

#### 4.2.1. *Ortho*-aminoanilides Pharmacokinetic Profile

**Figure 23:**
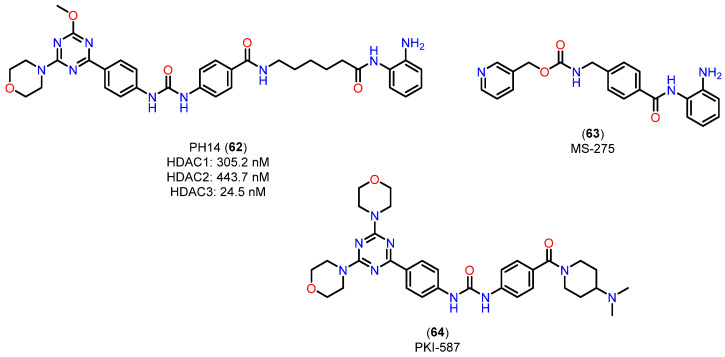
Chemical structures of PH14 (**62**), MS-275 (**63**), and PKI-587 (**64**).

In the study by Deng and colleagues [152], the compound PH14 (**62**), investigated for its inhibitory activity on CYP450 enzymes (Figure 23), displays a pharmacokinetic profile that suggests it may be a promising candidate for developing dual HDAC/PI3K inhibitors. This profile was characterized through assays evaluating its enzyme inhibition rates, cardiovascular safety, and metabolic stability, and included a comparative analysis with established compounds, such as MS-275 (**63**) [153], a benzamide-class HDAC inhibitor.

CYP450 enzyme inhibition by PH14 (**62**) was relatively low (less than 50% for enzymes 1A2, 2B6, 2C9, 2C19, and 3A4), with moderate inhibition of CYP2D6 (48.83%) at a concentration of 10 μM. This inhibition profile suggests that PH14 (**62**) is a weak CYP450 inhibitor, highlighting a potential drug interaction risk, particularly with CYP2D6, warranting caution in cases of co-administration. Additionally, the hERG channel inhibition assay, used to predict potential cardiovascular effects, showed an inhibition rate of 15.16% at a 30 μM concentration, indicating a low likelihood of adverse cardiac effects associated with PH14 (**62**) use. In terms of microsomal stability, PH14 (**62**) demonstrated comparable metabolic rates in liver microsomes from mice, rats, and humans, with half-lives of 26.93, 30.92, and 37.48 min, respectively. This result indicates moderate metabolic stability, and suggests potential for cross-species development, facilitating its assessment in preclinical studies. Compared to the HDAC inhibitor MS-275 (**63**) [153], a benzamide with high oral bioavailability (85%) and a favorable pharmacokinetic profile, PH14 (**62**) has a chemical structure similar to the PI3K inhibitor PKI-587 (**64**) (gedatolisib) [154]. PKI-587 (**64**) is known for its low plasma clearance and long half-life, characteristics that were also observed for PH14 (**62**) in mouse studies, where the t_1/2_ was 10 h and the AUC_(0-∞)_ was 2772 h·ng/mL at an intravenous dose of 1 mg/kg. These parameters suggest that PH14 (**62**), like PKI-587 (**64**), has a long half-life and low plasma clearance, which could support a favorable dosing regimen in terms of duration of action. On the other hand, compared to hydroxamic acids like vorinostat (**1**) [155], which exhibit high clearance and low oral bioavailability, PH14 (**62**) stands out with characteristics closer to benzamides, with potential for improved stability and systemic availability. This position it favorably compared to inhibitors with rapid clearance profiles, like hydroxamic acids, suggesting potential for development in oral formulations.

In summary, PH14 (**62**) exhibits a modest inhibitory profile on CYP450 enzymes, low potential for cardiac effects, and pharmacokinetic characteristics resembling PI3K inhibitors with prolonged half-life and low clearance. These findings provide a solid foundation for exploring PH14 (**62**) in combination therapies, particularly in dual HDAC/PI3K inhibition strategies, with potential therapeutic applications in oncology and diseases related to epigenetic metabolism.

#### 4.2.2. Mercaptoacetamides Pharmacokinetic Profile

**Figure 24:**
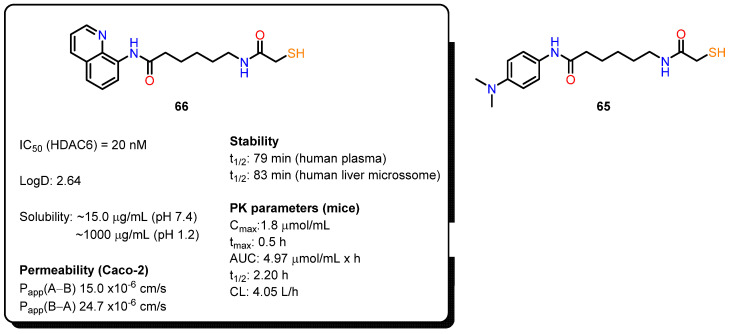
Chemical structures **65** and **66** with their PK parameters.

Compared to the extensive biological evaluation of hydroxamate-based HDACi, there has been limited investigation into the pharmacokinetics (PK), pharmacodynamics (PD), and pharmacological effects of mercaptoacetamide-based HDAC6 inhibitors in cancer and Alzheimer’s disease models. Recent studies [24,110] have focused on the ADME properties and PK/PD correlations of two key compounds: compound **65** (Figure 24), with an *N*,*N*-dimethylaminophenyl cap, and compound **66**, with a quinoline ring. Replacing the hydroxamate group with mercaptoacetamide was found to reduce molecular polarity while maintaining lipophilicity, with the dimethylamino and quinoline groups enhancing solubility in acidic environments, such as the stomach. Both compounds show permeability coefficients favorable for brain penetration, with compound **66** displaying a slightly superior PK profile and better plasma stability than compound **65**. In mouse models, both compounds were well tolerated and significantly increased histone acetylation levels, particularly in the brain and liver, supporting their blood–brain barrier penetration capabilities. Notably, compound **66**, with higher selectivity for HDAC6 over HDAC1, was effective in tubulin acetylation in glioma studies, inhibiting cell migration and invasion. Moreover, HDAC6 is a promising target for neurodegenerative diseases due to its modulation of key proteins. In Alzheimer’s models, compound **66** reduced β-amyloid (Aβ) and phosphorylated tau levels and increased dendritic spine density in transgenic mice, improving memory and learning deficits.

#### 4.2.3. Alkylhydrazides Pharmacokinetic Profile

**Figure 25:**
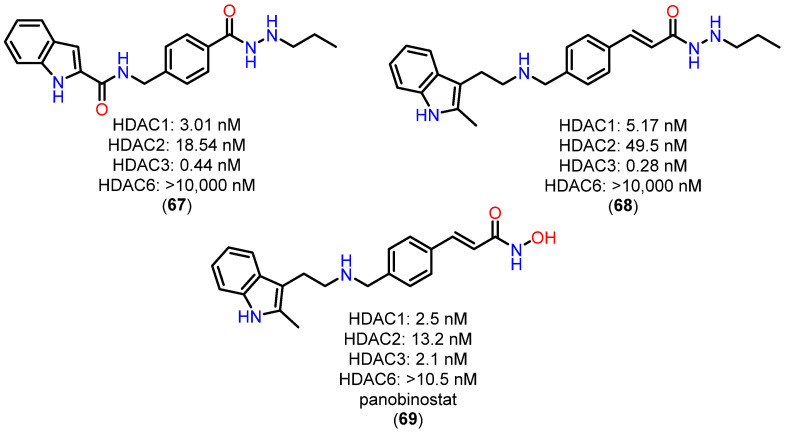
Chemical structures of **67**, **68** and panobinostat (**69**).

For the alkylhydrazide, the results highlight the remarkable pharmacokinetic (PK) profile of compound **67** compared to other HDACi, such as reference compound **68** and the hydroxamate panobinostat (**69**) (Figure 25). These findings underscore significant advantages that **67** is a promising candidate for clinical applications [119].

First, the analysis of C_max_ and AUC_0-inf_ demonstrates the clear superiority of **67**. At an oral dose of 20 mg/kg, the Cmax of **67** reached 38,800 ng/mL, which is 860 times higher than that of compound **68** (20 mg/kg) and 332 times higher than that of Panobinostat (**69**) (50 mg/kg). Similarly, the AUC_0-inf_ of **67** was 545 times greater than that of **68** and 1146 times greater than that of panobinostat (**69**). These results indicate that **67** possesses highly favorable PK characteristics, enabling significantly improved absorption and prolonged systemic exposure—critical factors for therapeutic success [119].

Another key pharmacokinetic parameter highlighting the superiority of **67** is its oral bioavailability (F%), which reached an impressive 112%, far surpassing compound **68** (19.8%) and panobinostat (**69**) (4.62%). This exceptional bioavailability reflects efficient absorption and suggests substantially reduced metabolic loss and pre-systemic clearance, challenges often encountered with hydroxamate-based HDACi [119].

The limited PK profiles of hydroxamate-based HDACi, such as panobinostat (**69**), often constrain their clinical application due to the need for high doses and complex therapeutic regimens. The significantly enhanced performance of **67** in this regard positions it as a promising alternative, potentially offering greater efficacy and convenience for patients. Moreover, the ability to achieve high plasma levels with oral administration is a crucial feature for improving treatment adherence, particularly in chronic or severe conditions like acute myeloid leukemia (AML) [119].

Thus, **67** addresses one of the key challenges in the development of novel HDACi: optimizing pharmacokinetic parameters to maximize efficacy while minimizing therapeutic drawbacks. The combination of high bioavailability, greater systemic exposure, and superior C_max_ and AUC values underscores **67** as a candidate capable of overcoming the limitations of currently available compounds, such as panobinostat (**69**).

These findings provide a robust foundation for advancing preclinical and clinical studies of **67**, aiming to explore its potential as a therapeutic agent for hematological diseases such as AML. Furthermore, the optimized PK parameters observed with **67** may serve as a model for the future development of more effective and clinically viable HDACi [119].

#### 4.2.4. 5-(Trifluoromethyl)-1,2,4-oxadiazole (TFMO) and 5-(Difluoromethyl)-1,3,4-oxadiazole (DFMO) Pharmacokinetic Profile

Unlike traditional hydroxamate-based inhibitors, which are often limited by poor pharmacokinetics and restricted central nervous system (CNS) penetration, TFMO and DFMO derivatives demonstrate significant structural and pharmacokinetic advantages. These characteristics make them particularly well-suited for targeting class IIa HDACs, addressing critical challenges associated with this subfamily of enzymes [156].

**Figure 26:**
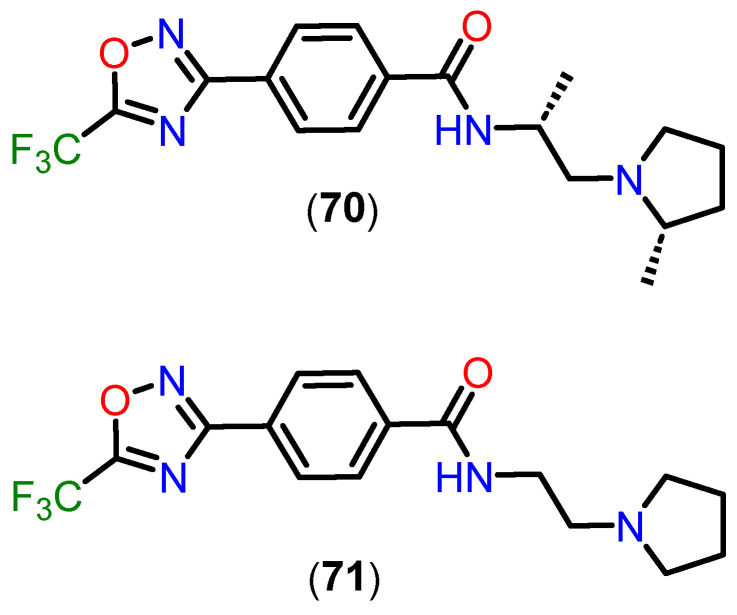
Chemical structures of **70** and **71**.

Among these compounds, TFMO-based HDAC inhibitor **70** (Figure 26) stands out for its favorable pharmacokinetic profile. It exhibits exceptional brain penetration, with brain-to-blood exposure ratios ranging from 1.3 to 8.7, depending on the dose. Notably, it achieves 100% oral bioavailability, a remarkable improvement over traditional HDAC inhibitors. This high bioavailability, combined with superior CNS distribution, underscores its potential for neurological applications. Structural optimizations, such as the introduction of a pyrrolidine group in derivative **71**, have further enhanced metabolic stability, as evidenced by improved clearance rates in mouse liver microsomes (Cl_int_ = 76 mL/min/kg). These modifications address the metabolic vulnerabilities observed in earlier analogs, enabling better systemic retention and efficacy [156].

Compound **70** also demonstrates rapid absorption, with a T_max_ of approximately 0.5 h. Interestingly, while blood concentrations of the compound increase proportionally with dose, brain concentrations show a supra-proportional rise, likely due to saturation of blood protein binding at higher doses. This results in greater availability of the free drug for CNS penetration, highlighting its suitability for central nervous system engagement. In vitro assays reveal a favorable unbound fraction in mouse blood (F% = 0.73) and brain homogenate (F% = 0.17), ensuring sufficient free drug to effectively engage targets in vivo.

The pharmacokinetic advantages of TFMO-based inhibitors are complemented by their selectivity and safety profile. Compound **70** exhibits over 100-fold selectivity for class IIa HDACs compared to class I and IIb isoforms, reducing the risk of off-target effects. Additionally, it shows minimal inhibition of cytochrome P450 enzymes and no significant interaction with hERG ion channels, indicating a low potential for adverse side effects. These features are critical for advancing the therapeutic potential of TFMO derivatives, particularly in addressing the limitations of broad-spectrum HDAC inhibitors [156].

The structural design of TFMO compounds also provides physicochemical benefits. By eliminating hydrogen bond donors present in hydroxamate-based inhibitors, TFMO derivatives exhibit improved lipophilicity and CNS permeability, further enhancing their pharmacokinetic profile. These modifications position TFMO-based inhibitors as promising candidates for diseases involving dysregulated HDAC activity [156].

The comprehensive pharmacokinetic and pharmacodynamic profile of compound **70** supports its advancement as a therapeutic candidate. Its ability to achieve high brain exposure, combined with excellent selectivity and safety, makes it particularly suitable for preclinical models of Huntington’s disease and other neurological disorders. By overcoming the challenges associated with hydroxamate-based inhibitors, TFMO derivatives represent a significant step forward in the development of targeted therapies for CNS conditions.

## 5. HDACi Approved for Clinical Use

**Figure 27:**
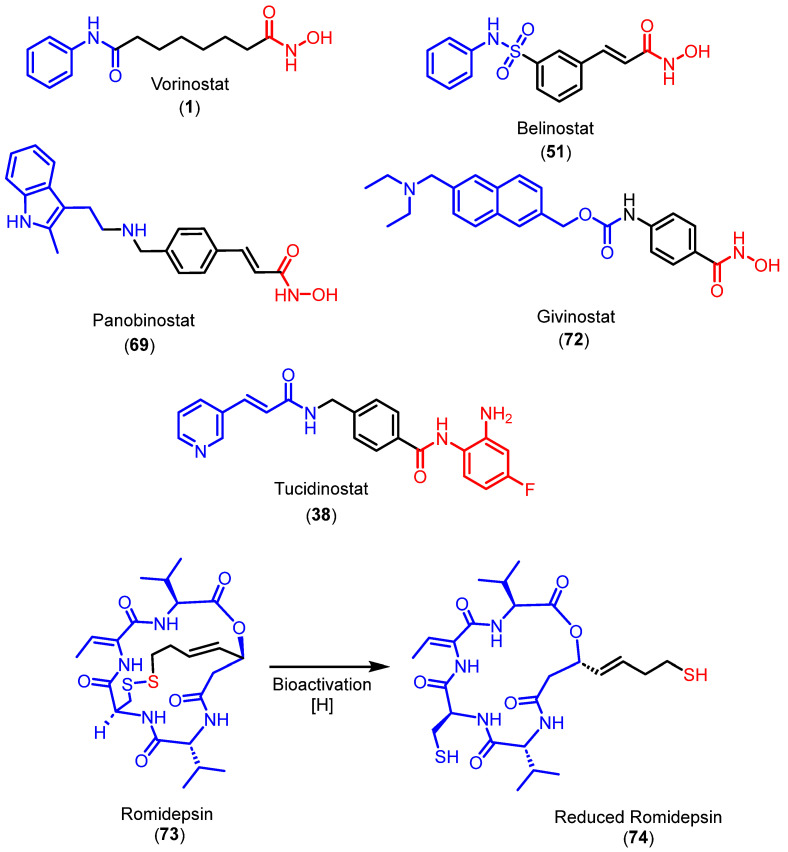
Chemical structures of approved drugs.

The majority of HDACi approved by the Food and Drug Administration (FDA) are used to treat cancer by interfering with epigenetic mechanisms and causing apoptosis, cell cycle disruption, and senescence. The majority of them contain hidroxamatos like ZBG [157]. Chemical structures of approved drugs are shown in Figure 27.

Among the FDA-approved drugs featuring hydroxamates as ZBGs are vorinostat (**1**), belinostat (**51**), panobinostat (**69**), and, more recently, givinostat (**72**), although in this case it was approved for the treatment of Duchenne Muscular Dystrophy (DMD). Romidepsin (**73**), on the other hand, also approved by the FDA, is a non-hydroxamate natural product that coordinates with the Zn^2+^ ion through a sulfur atom present in its structure following bioactivation. Tucidinostat (**38**) can also be highlighted as an inhibitor functionalized with an *ortho*-aminoanilide group, although, unlike the other cases, its approval was granted by the former China Food and Drug Administration (CFDA), now known as the National Medical Products Administration (NMPA) in China [23,24,157,158,159,160].

## 6. Conclusions

The development of HDAC inhibitors has undergone a remarkable transformation, moving beyond broad-spectrum inhibitors toward compounds with finely tuned selectivity and optimized pharmacokinetic properties. The choice of zinc-binding group plays a pivotal role in determining the affinity, selectivity, and metabolic stability of these inhibitors. Hydroxamic acids, while highly potent, face challenges related to rapid clearance and potential genotoxicity, prompting the exploration of alternative ZBGs such as *ortho*-aminoanilides, mercaptoacetamides, alkylhydrazides, and oxadiazoles.

Each of these alternative ZBGs presents unique advantages. *Ortho*-aminoanilides, for instance, demonstrate preferential inhibition of class I HDACs, particularly HDAC1, 2, and 3, which are closely linked to cancer progression. Mercaptoacetamides have emerged as promising selective inhibitors of HDAC6, a target implicated in neurodegenerative diseases and immune regulation. Alkylhydrazides and oxadiazole-based ZBGs offer novel binding mechanisms that not only enhance selectivity but also improve pharmacokinetic profiles, making them attractive candidates for drug development.

Beyond the selection of ZBGs, structural modifications in the cap group and linker region have also proven critical in modulating HDACi activity and selectivity. Exploiting subcavities within HDAC active sites—such as the foot pocket in HDAC1/2, the side pocket in HDAC8, and the lower pocket in HDAC4—has enabled the rational design of isoform-selective inhibitors. These advances are particularly important for minimizing off-target effects and reducing toxicity, which have been major limitations of first-generation HDACis.

Despite significant progress, challenges remain. One of the primary obstacles is achieving an optimal balance between potency, selectivity, and pharmacokinetics. Many potent inhibitors suffer from poor bioavailability or rapid metabolism, limiting their clinical applicability. Future research should focus on developing prodrug strategies, improving metabolic stability, and leveraging computational approaches to predict and optimize HDACi behavior in biological systems. Additionally, expanding the therapeutic scope of HDAC inhibitors beyond oncology—into areas such as neuroprotection, immunotherapy, and metabolic disorders—remains an exciting frontier.

Ultimately, the future of HDAC-targeted therapy lies in precision medicine, where selective inhibitors tailored to specific isoforms and disease contexts can maximize therapeutic benefits while minimizing adverse effects. Continued efforts in medicinal chemistry, structural biology, and translational research will be essential in unlocking the full potential of HDAC inhibitors as next-generation therapeutics.

## Figures and Tables

**Table 1:** Subcavities present in the active site of HDACs according to HDAC classes, with emphasis on subcavities present (green), absent (orange) and present in specific isoforms (purple).

Class	Acetate-Binding Cavity	Main Channel	Surface	Side Pocket	Lower Pocket	Foot Pocket
HDAC class I	Present	Present	Present	Present only in HDAC8	Absent	Present only in HDACs 1–3
HDAC class IIa	Present	Present	Present	Absent	Present	Absent
HDAC class IIb	Present	Present	Present	Absent	Absent	Absent
HDAC class IV	Present	Present	Present	Absent	Absent	Absent

**Table 2:** Overview of pharmacokinetic profile of main ZBGs.

ZBG	Metabolic Stability	Bioavailability	Additional Data
Hydroxamate	Accelerated glucuronidation	Low	-
*Ortho*-aminoanilides	Moderate	High	Weak CYP450 inhibitor and low hERG risks
Mercaptoacetamides	-	-	Favorable for brain penetration
Alkylhidrazides	Moderate	High	Reduced metabolic loss and pre-systemic clearance
TFMO and DFMO	-	High	High brain penetration and minimal CYP450 inhibition and hERG interactions

## Data Availability

No new data were created or analyzed in this study. Data sharing is not applicable to this article.
